# Phylogeny and diversity of *Bjerkandera* (Polyporales, Basidiomycota), including four new species from South America and Asia

**DOI:** 10.3897/mycokeys.79.63908

**Published:** 2021-04-26

**Authors:** Chao-Ge Wang, Josef Vlasák, Yu-Cheng Dai

**Affiliations:** 1 School of Ecology and Nature Conservation, Beijing Forestry University, Beijing 100083, China Beijing Forestry University Beijing China; 2 Biology Centre, Czech Academy of Sciences, Institute of Plant Mol. Biol., Branišovská 31, CZ-370 05 České Budějovice, Czech Republic Biology Centre, Czech Academy of Sciences České Budějovice Czech Republic

**Keywords:** Phylogeny, polypore, taxonomy, wood-decaying fungi

## Abstract

Four new species of *Bjerkandera*, viz. *B. ecuadorensis*, *B. fulgida*, *B. minispora*, and *B. resupinata***spp. nov.**, are described from tropical America and Asia. *B. ecuadorensis* is characterised by dark grey to black pore surface, a monomitic hyphal system, hyaline to yellowish-brown generative hyphae, and ellipsoid basidiospores measuring 3.9–4.5 × 2.7–3 μm. *B. fulgida* is distinguished from the other species in the genus by clay buff to pale brown and shiny pore surface. *B. minispora* is characterised by white tomentose pore mouth and small basidiospores measuring 3.1–4.2 × 2–2.8 μm. *B. resupinata* is characterised by resupinate basidiomata, pinkish buff to pale brownish pore surface, and ellipsoid to broadly ellipsoid basidiospores measuring 4.5–6 × 3.2–4.1 µm. All these new species grow on angiosperm trunks or rotten wood, and cause a white rot. The closely related taxa to four new species are discussed. An identification key to the ten accepted species of *Bjerkandera* is provided, and a phylogeny comprising all known *Bjerkandera* species is provided.

## Introduction

The genus *Bjerkandera* P. Karst. (Polyporales, Basidiomycota), typified by *B. adusta* (Willd.) P. Karst., was established by Karsten (1879). It is traditionally characterised by annual, effused-reflexed to pileate basidiomata, the presence of a dark resinous layer between context and tubes, grey to black pore surface, which contrasts with the pale cream context, a monomitic hyphal system with abundant clamps on generative hyphae, oblong ellipsoid to ellipsoid, hyaline, thin-walled basidiospores, and a white-rotting ecology (Murrill 1907; Ryvarden and Gilbertson 1993; Núñez and Ryvarden 2001; Westphalen et al. 2015; Cui et al. 2019). Based on the above morphological studies, *Bjerkandera* and *Gloeoporus* Mont. share some important characteristics, and both genera are confused about definition (Pilát 1937; Corner 1989; Motato-Vásquez et al. 2020). Also, *Tyromyces* P. Karst. is defined by white, annual, resupinate to pileate basidiomata, a mono-dimitic hyphal system with clamped generative hyphae, and a white-rotting ecology, so *Tyromyces* and *Bjerkandera* overlap in many characteristics (Ryvarden 1991; Pouzar 1966; Núñez and Ryvarden 2001). Due to these similarities, Pouzar (1966) considered *Bjerkandera* as subgenus of *Tyromyces* (Kotlaba and Pouzar 1964). Nevertheless, recent phylogenetic analyses have showed that *Bjerkandera*, *Gloeoporus*, and *Tyromyces* belong to different clades in the families Phanerochaetaceae Jülich (Syn. Bjerkanderaceae Jülich 1981), Irpicaceae Spirin & Zmitr., and Incrustoporiaceae Jülich, respectively (Binder et al. 2013; Floudas and Hibbett 2015; Justo et al. 2017; Jung et al. 2018; Viktor and Bálint 2018; Cui et al. 2019; Motato-Vásquez et al. 2020). Morphologically, *Tyromyces* differs in pale tubes not darkening upon drying, and *Gloeoporus* usually has a continuum hymenium (covering the dissepiments) and gelatinous tubes (Ryvarden and Gilbertson 1993), while species of *Bjerkandera* have distinct sterile dissepiments, and corky to hard corky tubes.

*Bjerkandera* is a common polypore genus that grows mostly on dead angiosperm wood and has a wide distribution around the world. Two species, *Bjerkandera adusta* (Willd.) P. Karst. and *B. fumosa* (Pers.) P. Karst., are well recognised in the northern hemisphere (Gilbertson and Ryvarden 1986; Jung et al. 2014; Ryvarden and Melo 2017; Cui et al. 2019). *Tyromyces atroalbus* (Rick) Rajchenb. was combined into *Bjerkandera* based on morphology and molecular phylogenetic analysis (Westphalen et al. 2015). Recently, two new species, *Bjerkandera albocinerea* Motato-Vásq., Robledo & Gugliotta and *B. centroamericana* Kout, Westphalen & Tomšovský, were described from the neotropics based on morphological characters and molecular data (Westphalen et al. 2015; Motato-Vásquez et al. 2020). Additionally, *B. mikrofumosa* Ryvarden was described from Venezuela without molecular data (Ryvarden 2016), but DNA sequences from this species were generated by Motato-Vásquez et al. (2020).

During a study on polypores collected from China, Ecuador, and Thailand, four unknown species of *Bjerkandera* were distinguished by both morphological and molecular data. They are described and illustrated in this study. In this study, nuclear ribosomal RNA genes were used to determine the phylogenetic position of the new species. Furthermore, an identification key to all the accepted species in the genus is provided.

## Materials and methods

### Morphological studies

The studied specimens are deposited in the herbaria of the Institute of Microbiology, Beijing Forestry University (**BJFC**), and the private herbarium of Josef Vlasák (**JV**), which will later be deposited at the National Museum Prague of Czech Republic (**PRM**). Morphological descriptions are based on field notes and herbarium specimens. Microscopic analyses follow Miettinen et al. (2018). In the description: **KOH** = 5% potassium hydroxide, **IKI** = Melzer’s reagent, **IKI**– = neither amyloid nor dextrinoid, **CB** = Cotton Blue, **CB**+ = cyanophilous in Cotton Blue, **CB**– = acyanophilous in Cotton Blue, **L** = arithmetic average of all spore length, **W** = arithmetic average of all spore width, **Q** = L/W ratios, and **n** = number of spores/measured from given number of specimens. Colour terms are cited from Anonymous (1969) and Petersen (1996).

### Molecular studies and phylogenetic analysis

A CTAB rapid plant genome extraction kit-DN14 (Aidlab Biotechnologies Co., Ltd, Beijing) was used to obtain DNA from dried specimens, and to perform the polymerase chain reaction (PCR) according to the manufacturer’s instructions with some modifications (Shen et al. 2019; Sun et al. 2020). Two DNA gene fragments – internal transcribed spacer (ITS) and large subunit nuclear ribosomal RNA gene (nLSU) – were amplified using the primer pairs ITS5/ITS4 and LR0R/LR7 (White et al. 1990; Hopple and Vilgalys 1999) (http://www.biology.duke.edu/fungi/mycolab/primers.htm). The PCR procedures for ITS and nLSU followed Zhao et al. (2013) in the phylogenetic analyses. DNA sequencing was performed at Beijing Genomics Institute and the newly-generated sequences were deposited in GenBank (Sayers et al. 2021). Sequences generated for this study were aligned with additional sequences downloaded from GenBank using BioEdit (Hall 1999) and ClustalX (Thompson et al. 1997). The final ITS and nLSU datasets were subsequently aligned using MAFFT v.7 under the E-INS-i strategy with no cost for opening gaps and equal cost for transformations (command line: mafft –genafpair –maxiterate 1000) (Katoh and Standley 2013) and visualised in BioEdit (Hall 1999).

In this study, nuclear ribosomal RNA genes were used to determine the phylogenetic position of the new species. The sequence alignment was deposited at TreeBase (submission ID 27872). Sequences of *Tyromyces chioneus* (Fr.) P. Karst, obtained from GenBank, was used as outgroup (Westphalen et al. 2015).

The phylogenetic analyses followed the approach of Han et al. (2016) and Zhu et al. (2019). Maximum parsimony (MP), maximum likelihood (ML), and Bayesian inference (BI) analyses were conducted for the datasets of ITS and nLSU sequences. The best-fit evolutionary model was selected by hierarchical likelihood ratio tests (hLRT) and Akaike Information Criterion (AIC) in MrModeltest 2.2 (Nylander 2004) after scoring 24 models of evolution in PAUP* version 4.0b10 (Swofford 2002).

The MP topology and bootstrap values (MP-BS) obtained from 1000 replicates were computed in PAUP* version 4.0b10 (Swofford 2002). All characters were equally weighted, and gaps were treated as missing. Trees were inferred using the heuristic search option with TBR branch swapping and 1000 random sequence additions. Max-trees were set to 5,000 branches of zero length were collapsed, and all parsimonious trees were saved. Descriptive tree statistics tree length (TL), composite consistency index (CI), retention index (RI), rescaled consistency index (RC), and homoplasy index (HI) were calculated for each maximum parsimonious tree (MPT) generated. Sequences were also analysed using Maximum Likelihood (ML) with RAxML-HPC2 through the CIPRES Science Gateway (www.phylo.org; Miller et al. 2009). Branch support (BT) for ML analysis was determined by 1000 bootstrap replicates.

Bayesian phylogenetic inference and Bayesian posterior probabilities (BPP) were computed with MrBayes 3.1.2 (Ronquist and Huelsenbeck 2003). Four Markov chains were run for 3,500,000 generations until the split deviation frequency value was less than 0.01 and trees were sampled every 100 generations. The first 25% of the sampled trees were discarded as burn-in and the remaining ones were used to reconstruct a majority rule consensus and calculate Bayesian posterior probabilities (BPP) of the clades.

Branches that received bootstrap support for maximum parsimony (≥ 75% MP-BT), maximum likelihood (≥75% (ML-BS)), and Bayesian posterior probabilities (≥ 0.95BPP) were considered as significantly supported.

## Results

### Phylogeny

**Figure 1.**
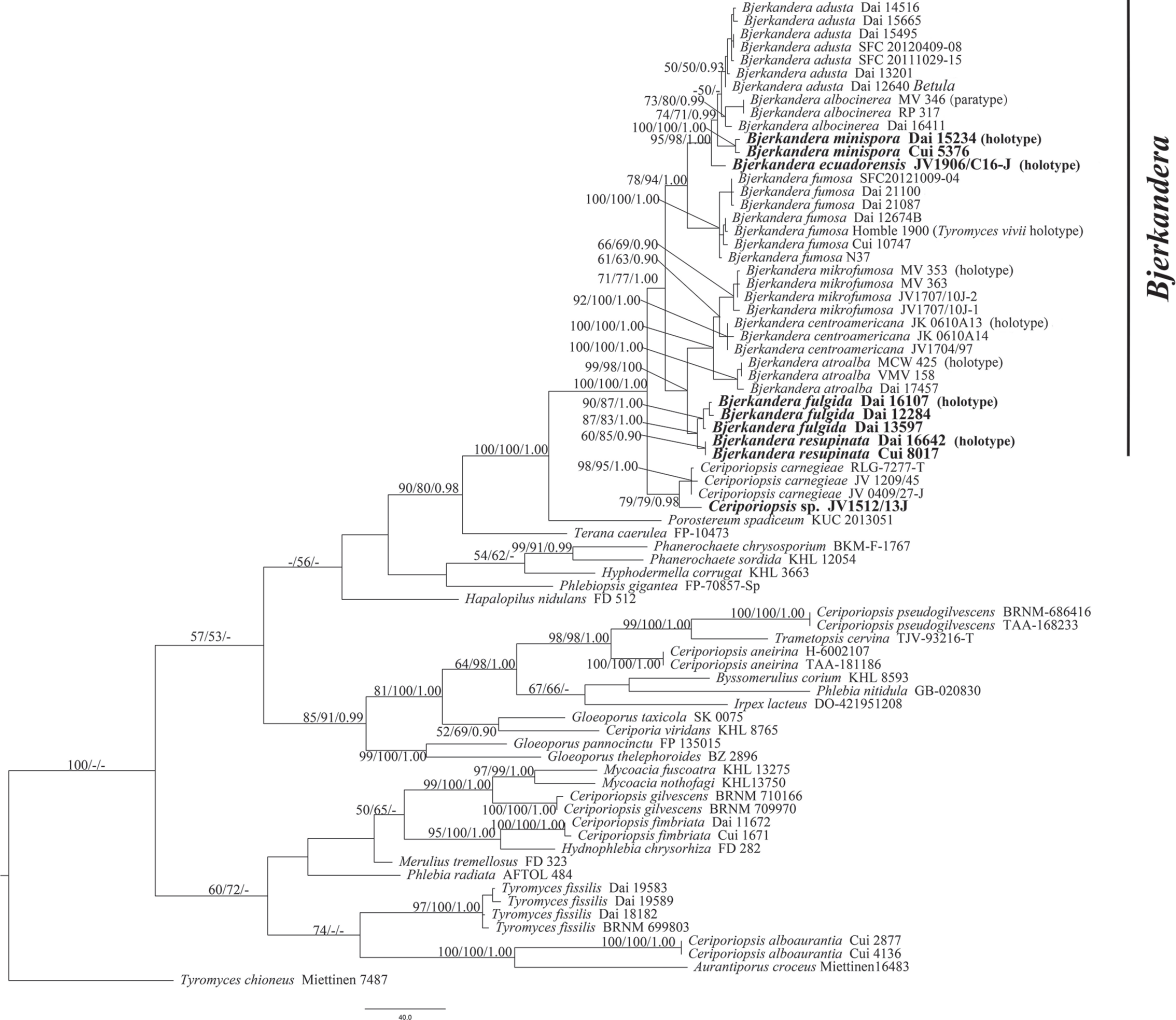
Phylogeny of *Bjerkandera* and related species generated by maximum parsimony analysis, based on combined ITS and nLSU sequences. Bootstrap support for maximum parsimony (MP), maximum likelihood (ML), and Bayesian posterior probabilities: (BPP) ≥ 50% (MP-BT), 50% (ML-BS) and 0.90 (BPP) are given in relation to the branches.

The combined ITS and nLSU dataset contained sequences from 75 specimens, comprising a total of 40 species (Table 1). The dataset had an aligned length of 2158 characters, of which 1410 (65%) characters are constant, 208 (0.1%) are variable and parsimony-uninformative and 540 (25%) are parsimony informative. Maximum parsimony analysis yielded eleven equally-parsimonious tree (TL = 2701, CI = 0.439, RI = 0.751, RC = 0.330, HI = 0.561), and a strict consensus tree of these trees is shown in Fig. 1. The best model-fit applied in the Bayesian analysis was GTR+I+G, lset nst = 6, rates = invgamma, and prset statefreqpr = dirichlet (1, 1, 1, 1). Bayesian analysis resulted in the nearly congruent topology with an average standard deviation of split frequencies = 0.006804 to MP and ML analysis, and thus only the MP tree was provided.

**Table 1.** Information on the sequences used in this study. New sequences are shown in bold.

Species	Specimen number	Countries	GenBank accession numbers	
			ITS	LSU
*Aurantiporus croceus*	Miettinen-16483	Malaysia	KY948745	KY948901
***Bjerkandera adusta***	**Dai 14516**	**China**	**MW507097**	**MW520204**
*** adusta ***	**Dai 15665**	**China**	**MW507098**	**MW520205**
***B. adusta***	**Dai 15495**	**China**	**MW507099**	–
*B. adusta*	SFC20120409-08	Rep. Korea	KJ704814	KJ704829
*B. adusta*	SFC20111029-15	Rep. Korea	KJ704813	KJ704828
***B. adusta***	**Dai 13201**	**France**	**MW507100**	**MW520206**
***B. adusta***	**Dai 12640**	**Finland**	**MW507101**	–
*B. albocinerea*	MV 346	Brazil	MH025421	MH025421
*B. albocinerea*	RP 317	Brazil	MH025420	–
***B. albocinerea***	**Dai 16411**	**USA**	**MW507102**	**MW520207**
*B. atroalba*	MW 425	Brazil	KT305930	KT305930
*B. atroalba*	MV 158	Brazil	KT305932	KT305932
***B. atroalba***	**Dai 17457**	**Brazil**	**MW507103**	**MW520208**
*B. centroamericana*	JK0610/A13	Mexico	KT305934	KT305934
*B. centroamericana*	JK0610/A7	Mexico	KT305933	KT305933
***B. centroamericana***	**JV1704/97**	**Costa Rica**	**MW507104**	–
***B. ecuadorensis***	**JV1906/C16-J**	**Ecuador**	**MW507105**	–
***B. fulgida***	**Dai 16107**	**China**	**MW507106**	**MW520209**
***B. fulgida***	**Dai 12284**	**China**	**MW507107**	–
***B. fulgida***	**Dai 13597**	**China**	**MW507108**	**MW520210**
*B. fumosa*	SFC20121009-04	Rep. Korea	KJ704824	KJ704839
***B. fumosa***	**Dai 21100**	**China**	**MW507109**	**MW520211**
***B. fumosa***	**Dai 21087**	**China**	**MW507110**	–
***B. fumosa***	**Cui 10747**	**China**	**MW507111**	**MW520212**
***B. fumosa***	**Dai 12674B**	**Finland**	**MW507112**	**MW520213**
*B. fumosa*	N37	Latvia	FJ903376	–
*B. fumosa*	Homble 1900	Norway	KF698740	KF698751
*B. mikrofumosa*	MV 353	Brazil	MH025416	MH025416
*B. mikrofumosa*	MV 363	Brazil	MH023526	MH023526
***B. mikrofumosa***	**JV1707/10J-1**	**Costa Rica**	**MW507113**	–
***B. mikrofumosa***	**JV1707/10J-2**	**Costa Rica**	**MW507114**	–
***B. minispora***	**Dai 15234**	**China**	**MW507115**	**MW520214**
***B. minispora***	**Cui 5376**	**China**	**MW507116**	**MW520215**
***B. resupinata***	**Dai 16642**	**Thailand**	**MW507117**	**MW520216**
***B. resupinata***	**Cui 8017**	**China**	**KU509526**	–
*Byssomerulius corium*	KHL 8593	–	AY463389	AY586640
*Ceriporia viridans*	KHL 8765	–	AF347109	AF347109
*Ceriporiopsis alboaurantia*	Cui 4136	China	KF845955	KF845948
*C. alboaurantia*	Cui 2877	China	KF845954	KF845947
*C. aneirina*	TAA 181186	Estonia	FJ496683	FJ496704
*C. aneirina*	H 6002107	Finland	FJ496682	FJ496705
* carnegieae *	RLG-7277-T	USA	KY948792	KY948854
*C. carnegieae*	JV1209/45	USA	KX081134	–
***C. carnegieae***	**JV0407/27-J**	**USA**	**MW507122**	–
*C. fimbriata*	Dai 11672	China	KJ698633	KJ698637
*C. fimbriata*	Cui 1671	China	KJ698634	KJ698638
*C. gilvescens*	BRNM 710166	Czech	FJ496684	FJ496720
*C. gilvescens*	BRNM 709970	Czech	EU546104	FJ496721
*C. pseudogilvescens*	BRNM 686416	Slovakia	FJ496679	FJ496703
*C. pseudogilvescens*	TAA 168233	Estonia	FJ496673	FJ496702
***Ceriporiopsis* sp.**	**JV1512/13-J**	**Costa Rica**	**MW507118**	–
*Gloeoporus taxicola*	SK 0075	Sweden	JX109847	JX109847
*G. pannocinctus*	FP 135015	USA	MG572755	MG572739
*G. thelephoroides*	BZ 2896	Belize	MG572757	MG572741
*Hapalopilus nidulans*	FD-512	USA	KP135419	–
*Hydnophlebia chrysorhiza*	FD-282	USA	KP135338	KP135217
*Hyphodermella corrugata*	KHL 3663	Norway	EU118630	EU118630
*Irpex lacteus*	DO 421951208	Sweden	JX109852	JX109852
*Merulius tremellosus*	FD-323	USA	–	KP135231
*Mycoacia fuscoatra*	KHL 13275	Estonia	JN649352	JN649352
*M. nothofagi*	KHL 13750	France	GU480000	GU480000
*Phanerochaete chrysosporium*	BKM-F-1767	–	HQ188436	GQ470643
*P. sordida*	KHL 12054	Norway	EU118653	EU118653
*Phlebia nitidula*	GB 020830	Sweden	EU118655	EU118655
*P. radiata*	AFTOL 484	–	AY854087	AF287885
*Phlebiopsis gigantea*	FP-70857-Sp	USA	KP135390	KP135272
*Porostereum spadiceum*	KUC 2013051	Rep. Korea	KJ668473	KJ668325
*Terana caerulea*	FP 10473	USA	KP134980	KP135276
*Trametopsis cervina*	TJV 93216 T	USA	JN165020	JN164796
*Tyromyces chioneus*	Miettinen 7487	Finland	HQ659244	HQ659244
***T. fissilis***	**Dai 18182**	**China**	**MW507119**	**MW520217**
***T. fissilis***	**Dai 19583**	**China**	**MW507120**	**MW520218**
*T. fissilis*	BRNM 699803	Czech	HQ728292	HQ729002
***T. fissilis***	**Dai 19589**	**China**	**MW507121**	–

In our phylogeny (Fig. 1), the genus *Bjerkandera* was supported as a monophyletic clade, which was consistent with previous studies on monophyly nature of *Bjerkandera* (Westphalen et al. 2015; Motato-Vásquez et al. 2020). *Bjerkandera ecuadorensis*, *B. fulgida*, *B. minispora*, and *B. resupinata* were nested within the *Bjerkandera* forming four distinct lineages (95/98/1.00, 90/87/1.00, 100/100/1.00, and 60/85/0.90 respectively).

### Taxonomy

#### Bjerkandera ecuadorensis

Taxon classificationFungiPolyporalesMeruliaceae

Y.C. Dai, Chao G. Wang & Vlasák sp. nov.

36FA3096-FC4F-5848-A8CE-08D7C16C0462

538578

Figs 2 , 3

##### Diagnosis.

*Bjerkandera ecuadorensis* is characterised by grey to dark-brown pore surface, tiny pores (7–9 per mm), and ellipsoid basidiospores measuring 3.9–4.5 × 2.7–3 μm.

##### Type.

Ecuador, Pichincha Province, volcan Pasochoa, 3300 m, VI. 2019, J. Vlasák Jr. JV 1906/C16-J (holotype in PRM, isotypes in JV and BJFC032992).

##### Etymology.

*Ecuadorensis* (Lat.): referring to the species being found in Ecuador.

##### Basidiomata.

Annual, pileate, soft corky, without odor or taste when fresh, becoming corky when dry, projecting up to 4 cm, 5 cm wide and 1.3 mm thick at base. Pileal surface pinkish-buff to buff, glabrous, faintly zonate, margin blunt. Pore surface grey to dark-brown, becoming almost black when touched or bruised; sterile margin distinct, up to 2 mm wide; pores round to angular, 7–9 per mm; dissepiments thin, entire. Context buff-yellow, slightly fibrous to corky, up to 1 mm thick. Tubes concolorous with the pore surface and darker than context, corky, up to 0.3 mm long, and with a distinct dark line between tubes and context.

**Figure 2.**
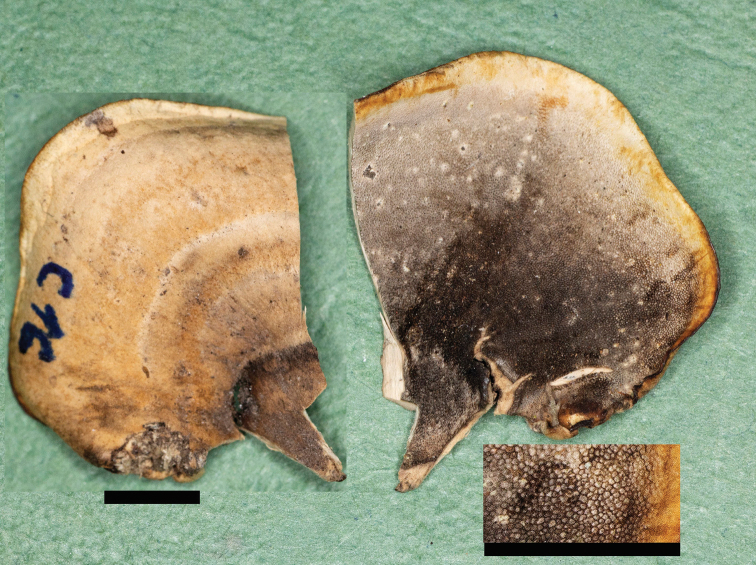
Pileal surface and pore surface of *Bjerkandera ecuadorensis* (holotype, JV 1906/C16-J). Scale bars: 4 mm.

##### Hyphal structure.

Hyphal system monomitic; generative hyphae with clamp connections, smooth, hyaline to yellowish-brown, CB+, IKI–; tissues becoming dark in KOH.

##### Context.

Generative hyphae thick-walled with a wide lumen, occasionally branched, densely compacted, and more or less regularly arranged to loosely interwoven, up to 3.8–6 μm in diam.

**Figure 3.**
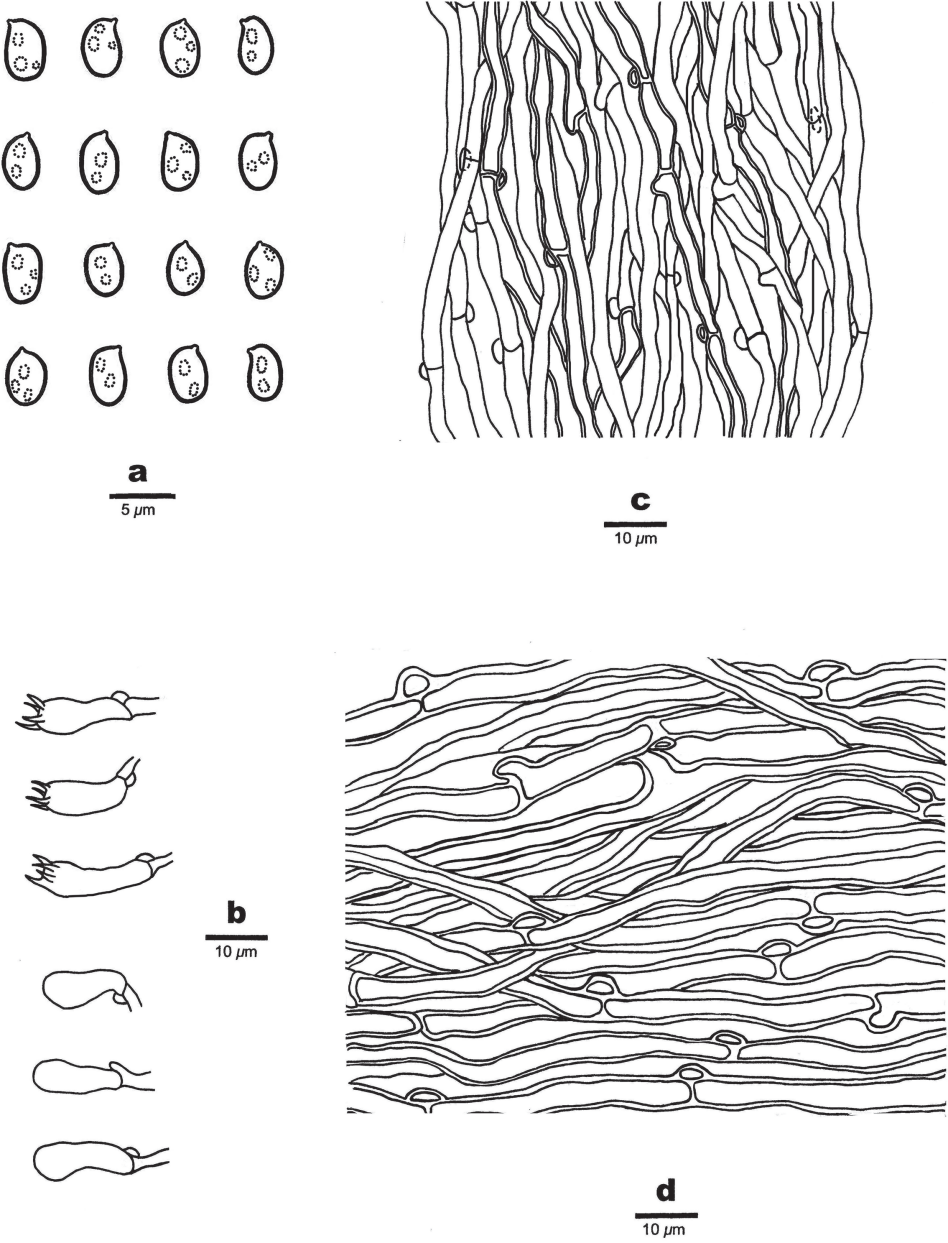
Microscopic structures of *Bjerkandera ecuadorensis* (holotype, JV 1906/C16-J) **a** basidiospores **b** basidia and basidioles **c** hyphae from trama **d** hyphae from context.

##### Tubes.

Generative hyphae thin- to slightly thick-walled, rarely branched, subparallel along the tubes to loosely interwoven, 2.5–3.8 μm in diam. Cystidia and cystidioles absent. Basidia clavate to barrel-shaped, with four sterigmata and a basal clamp connection, 13–14.5 × 4.5–5.5 µm; basidioles of similar shape to basidia, but smaller.

##### Basidiospores.

Ellipsoid, hyaline, thin-walled, smooth, often with one or more guttules, CB–, IKI–, (3.8–)3.9–4.5 × 2.7–3 µm, L = 4.09 μm, W = 2.86 μm, Q = 1.43 (n = 30/1).

##### Remarks.

*Bjerkandera ecuadorensis* is characterised by grey to dark-brown pore surface, small pores (7–9 per mm), hyaline to yellowish-brown generative hyphae, and ellipsoid basidiospores measuring 3.9–4.5 × 2.7–3 μm. Morphologically, *Bjerkandera ecuadorensis* is similar to *B. minispora* in having pinkish-buff to buff pileal surface and round to angular pores (6–9 per mm), but the latter has buff-yellow pore surface and smaller basidiospores (3.1–4.2 × 2–2.8 μm). *Bjerkandera adusta* resembles *B. ecuadorensis* by having grey to dark-brown pore surface, distinct sterile margin, but the former has short-cylindric to subellipsoid and bigger basidiospores (4.5–6 × 2.5–3.5 μm, Ryvarden and Melo 2017).

#### Bjerkandera fulgida

Taxon classificationFungiPolyporalesMeruliaceae

Y.C. Dai & Chao G. Wang sp. nov.

24FE9E10-8D5D-5E3E-BA33-02FCB1065DFD

838579

Figs 4 , 5

##### Diagnosis.

*Bjerkandera fulgida* is characterised by the clay buff to pale brown and shiny pore surface, and ellipsoid to broadly ellipsoid basidiospores measuring 3.9–4.5 × 2.8–3.3 μm.

##### Type.

China. Hainan Province, Lingshui County, Diaoluoshan Forest Park, 18°42'N, 109°49'E, rotten angiosperm wood, 13.XI.2015, Y.C. Dai 16107 (holotype BJFC020200).

##### Etymology.

*Fulgida* (Lat.): referring to the species having the shiny pore surface.

##### Basidiomata.

Annual, effused-reflexed, soft corky, without odor or taste when fresh, becoming corky upon drying, resupinating up to 5.5 cm long, 3 cm wide and 1.3 mm thick, with a pileal projection up to 0.6 cm, 2.3 cm wide and 1.3 mm thick at base. Pileal surface pinkish buff to clay-buff, glabrous and faintly zonate when dry; margin acute. Pore surface clay-buff to pale brown, bruised part becoming dark brown to black when dry, shiny; sterile margin up to 2 mm wide; pores round or sometimes angular, 6–8 per mm; dissepiments thin, entire. Context pale cream, slightly fibrous to corky, up to 0.5 mm thick. Tubes concolorous with the pore surface, darker than context, corky, up to 0.8 mm long, with a distinct dark line between tubes and context.

**Figure 4.**
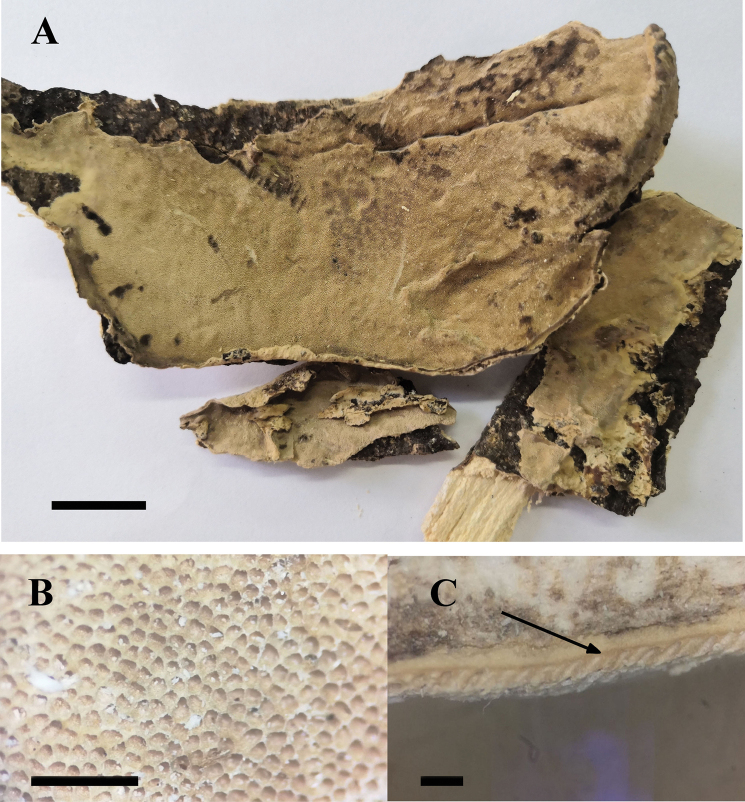
*Bjerkandera fulgida* (holotype, Y.C. Dai 16107) **A** basidiomata **B** poroid surface detail **C** a dark line between tubes and context. Scale bars: 1 cm (**A**); 1 mm (**B, C**).

##### Hyphal structure.

Hyphal system monomitic; generative hyphae with clamp connections, smooth, hyaline to yellowish, CB+, IKI–; tissues becoming dark in KOH.

##### Context.

Hyphae thick-walled with a wide lumen, occasionally branched, loosely interwoven, 3–5 μm in diam.

##### Tubes.

Hyphae thin- to slightly thick-walled, frequently branched, agglutinated and loosely interwoven, 2.5–3.5 μm in diam. Cystidia and cystidioles absent. Basidia clavate to more or less pyriform, with four sterigmata and a basal clamp connection, 10–12 × 4–5.5 μm; basidioles of similar shape to basidia, but smaller. Crystals present among hymenium.

**Figure 5.**
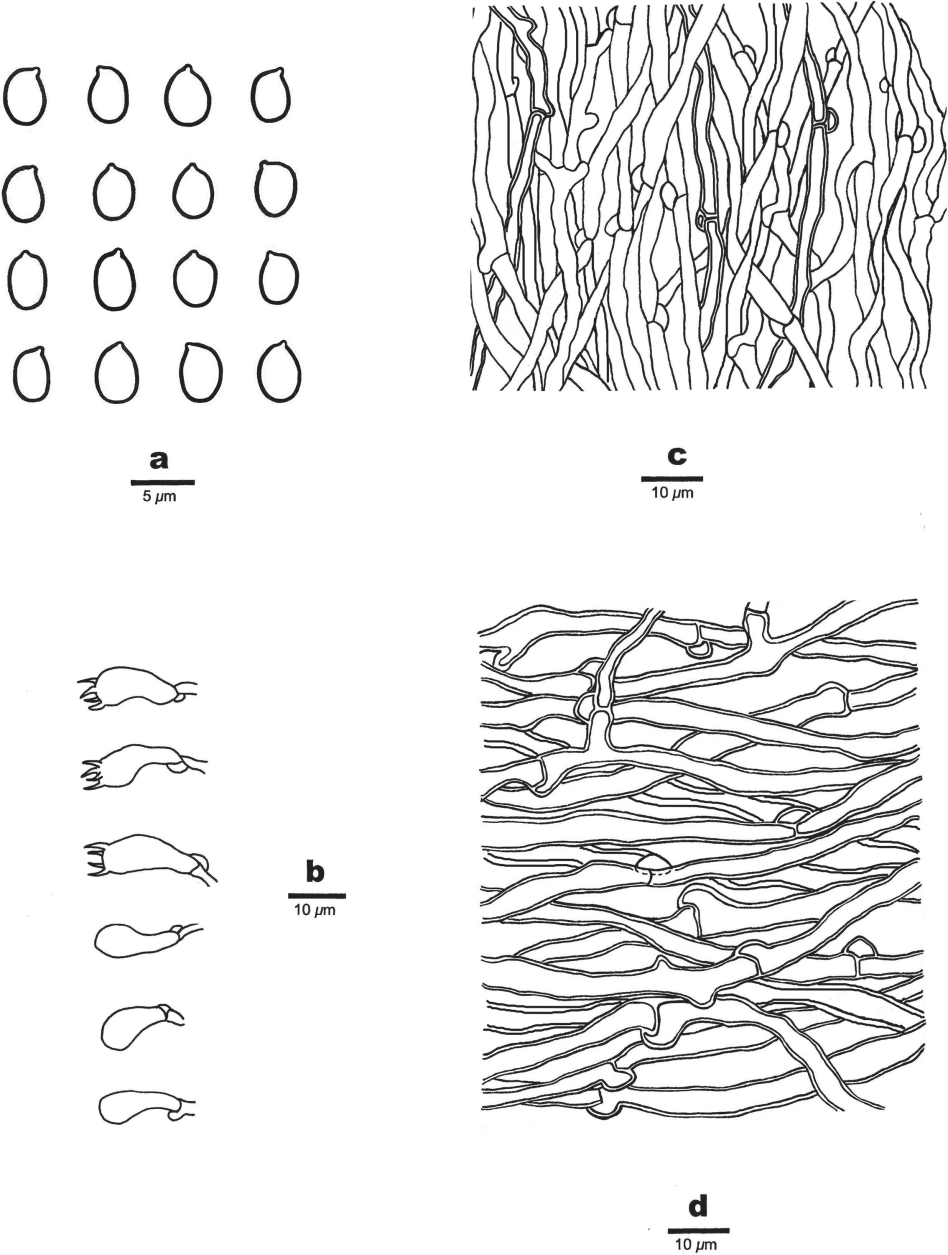
Microscopic structures of *Bjerkandera fulgida* (holotype, Y.C. Dai 16107) **a** basidiospores **b** basidia and basidioles **c** hyphae from trama **d** hyphae from context.

##### Basidiospores.

Ellipsoid to broadly ellipsoid, hyaline, thin-walled, smooth, CB–, IKI–, (3.8–)3.9–4.5 × (2.6–)2.8–3.3(–3.4) µm, L = 4.21 μm, W = 3.02 μm, Q = 1.37–1.43 (n = 90/3).

##### Additional specimens (paratypes) examined.

China. Yunnan Province, Jinghong, Sanchahe Nature Reserve, 22°09'N, 100°51'E, fallen angiosperm trunk, 24. VI. 2011, Y.C. Dai 12284 (BJFC010566); Xishuangbanna Tropical Botanical Garden, fallen angiosperm trunk, 21°55'N, 101°15'E, 21.X.2013, Y.C. Dai 13597 (BJFC015059).

##### Remarks.

*Bjerkandera fulgida* is characterised by the resupinate to effused-reflexed basidiomata, clay buff to pale brown and shiny pore surface, and ellipsoid to broadly ellipsoid basidiospores measuring 3.9–4.5 × 2.8–3.3 μm. Phylogenetically, *Bjerkandera resupinata* nests in a sister clade to *B. fulgida* (Fig. 1), also having morphological similarities, as the pore surface coloration and presence of branched hyphae in the tubes. However, *B. resupinata* differs in having resupinate basidiomata, larger pores (4–6 per mm), and basidiospores measuring 4.5–6 × 3.2–4.1 μm.

#### Bjerkandera minispora

Taxon classificationFungiPolyporalesMeruliaceae

Y.C. Dai & Chao G. Wang sp. nov.

17FA6080-8257-55F4-A6A4-8B2E7F7976DB

838580

Figs 6 , 7

##### Diagnosis.

The tiny pores (6–9 per mm), and ellipsoid small basidiospores measuring 3.1–4.2 × 2–2.8 μm set this species apart from others in *Bjerkandera*.

##### Type.

China. Hainan Province, Wuzhishan County, Wuzhishan Nature Reserve, 18°54'N, 109°42'E, fallen angiosperm trunk, 31. V. 2015, Y.C. Dai 15234 (holotype BJFC019345).

##### Etymology.

*Minispora* (Lat.): referring to the species having small basidiospores.

##### Basidiomata.

Annual, pileate, solitary or imbricate, soft corky, without odor or taste when fresh, becoming corky when dry. Pilei flabelliform, projecting up to 4 cm, 5 cm wide and 3 mm thick at base. Pileal surface pinkish-buff to buff, becoming dark when touched, velutinate to glabrous, azonate; margin a bit acute. Pore surface buff-yellow, ash-grey to pale brown when dry, touched or bruised parts becoming almost black; sterile margin distinct, up to 1.5 mm wide; pores tiny, round to angular, 6–9 per mm; pores mouth sometimes with white tomentum; dissepiments thin, entire to lacerate. Context cream to pinkish-buff, corky, up to 2 mm thick. Tubes concolorous with the pore surface, darker than context, corky, up to 1 mm long, with a distinct dark line between tubes and context.

**Figure 6.**
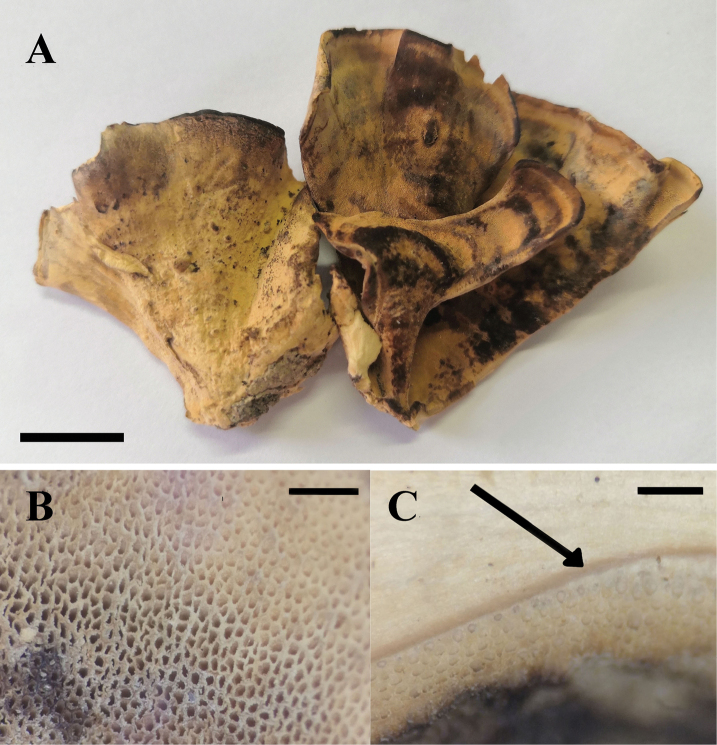
*Bjerkandera minispora* (holotype, Y.C. Dai 15234) **A** basidiomata **B** poroid surface detail **C** a dark line between tubes and context. Scale bars: 1 cm (**A**); 1 mm (**B, C**).

##### Hyphal structure.

Hyphal system monomitic; generative hyphae with clamp connections, smooth, hyaline to pale yellow, CB+, IKI–; tissues becoming dark in KOH.

##### Context.

Generative hyphae thick-walled with a wide lumen, moderately branched, loosely interwoven, 3.5–6 μm in diam.

**Figure 7.**
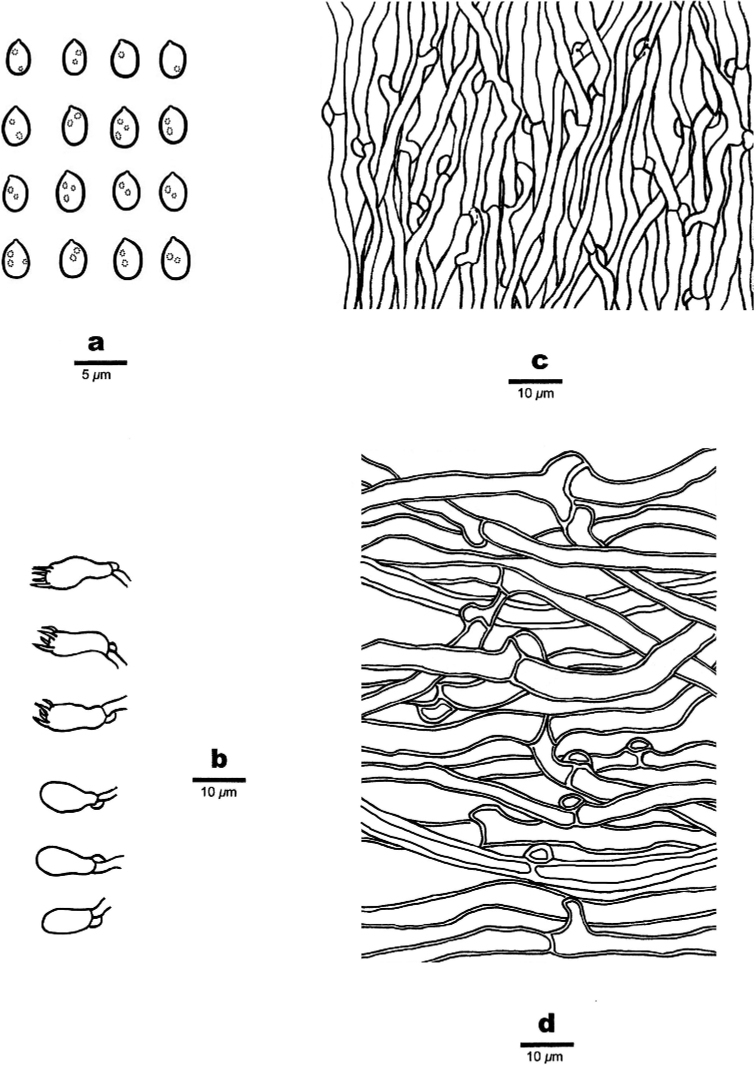
Microscopic structures of *Bjerkandera minispora* (holotype, Y.C. Dai 15234) **a** basidiospores **b** basidia and basidioles **c** hyphae from trama **d** hyphae from context.

##### Tubes.

Generative hyphae thin-walled, frequently branched, agglutinated and loosely interwoven, 2.5–3.5 μm in diam. Cystidia and cystidioles absent. Basidia clavate, sometimes with an intermediate constriction, with four sterigmata and a basal clamp connection, 9.5–11.5 × 4–5 μm; basidioles of similar shape to basidia, but smaller.

##### Basidiospores.

Oblong-ellipsoid to ellipsoid, hyaline, thin-walled, smooth, often with one or more guttules, CB–, IKI–, (3–)3.1–4.2(–4.8) × 2–2.8(–3) µm, L = 3.64 μm, W = 2.4 μm, Q = 1.49–1.54 (n = 60/2).

##### Additional specimen (paratype) examined.

China. Hainan Province, Wuzhishan County, Wuzhishan Nature Reserve, 18°54'N, 109°42'E, fallen angiosperm trunk, 24. XI. 2007, B.K. Cui 5376 (BJFC003417).

##### Remarks.

The buff-yellow pore surface, darkening when touched or bruised, the small pores (6–9 per mm) sometimes with white tomentum, and the ellipsoid small basidiospores (3.1–4.2 × 2–2.8 μm) set this species apart from others in *Bjerkandera*. *Bjerkandera albocinerea* resembles *B. minispora* by oblong-ellipsoid to ellipsoid basidiospores, but the former has sordid white fresh pileal surface, and dark brownish grey pore surface (Motato-Vásquez et al. 2020). *Bjerkandera ecuadorensis* is similar to *B. minispora* in having pinkish-buff to buff pileal surface and round to angular pores (6–9 per mm), but the former has grey to dark-brown pore surface and bigger basidiospores measuring 3.9–4.5 × 2.7–3 μm.

#### Bjerkandera resupinata

Taxon classificationFungiPolyporalesMeruliaceae

Y.C. Dai & Chao G. Wang sp. nov.

FBA4335A-2E14-5A6A-A4C7-0C4B69913A1F

838581

Figs 8 , 9

##### Diagnosis.

Differs from other species of *Bjerkandera* by resupinate basidiomata.

##### Type.

Thailand. Chiang Rai, Doi Mae Salong, rotten angiosperm trunk, 22. VII. 2016, Y.C. Dai 16642 (holotype BJFC022752).

##### Etymology.

*Resupinata* (Lat.): referring to the species having resupinate basidiomata.

##### Basidiomata.

Annual, resupinate, adnate, soft corky, without odor or taste when fresh, becoming corky when dry, up to 6 cm long, 2 cm wide, 0.5 mm thick at base. Pore surface pinkish buff to pale brownish when dry, becoming dark grey in bruised parts; sterile margin distinct, thinning out, somewhat incised, up to 3 mm wide; pores round to angular, 4–6 per mm; dissepiments thin, entire to lacerated. Subiculum pale cream, slightly fibrous to corky, up to 0.2 mm thick. Tubes concolorous with the pore surface, darker than the subiculum, corky, up to 0.3 mm long, with a distinct dark line between tubes and subiculum.

**Figure 8.**
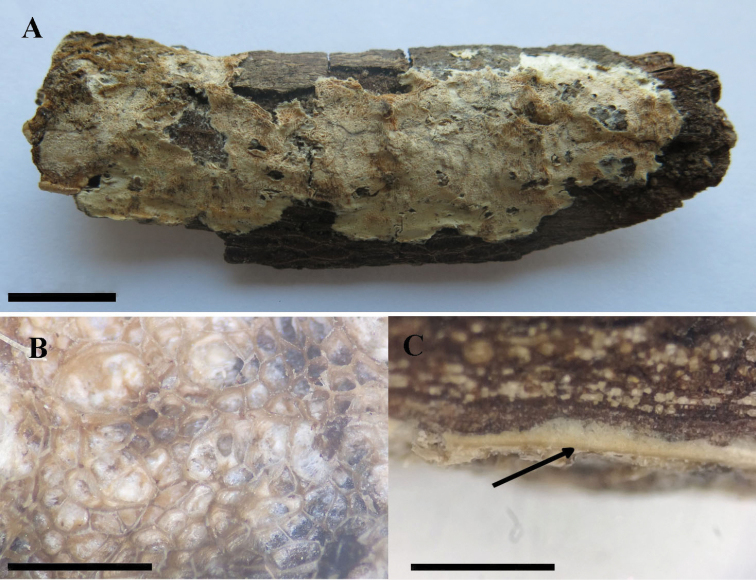
*Bjerkandera resupinata* (holotype, Y.C. Dai 16642) **A** basidiomata **B** poroid surface detail **C** a dark line between tubes and subiculum. Scale bars: 1 cm (**A**); 1 mm (**B, C**).

##### Hyphal structure.

Hyphal system monomitic; generative hyphae with clamp connections, smooth, hyaline to yellowish, CB+, IKI–; tissues becoming dark in KOH.

##### Subiculum.

Generative hyphae thick-walled with a wide lumen, rarely branched, loosely interwoven, 4–5 μm in diam.

##### Tubes.

Generative hyphae thin- to slightly thick-walled, frequently branched, loosely interwoven, 2.7–3.8 μm in diam. Cystidia and cystidioles absent. Basidia clavate, with four sterigmata and a basal clamp connection, 14–16 × 5–6.5 μm; basidioles in shape similar to basidia, but smaller.

**Figure 9.**
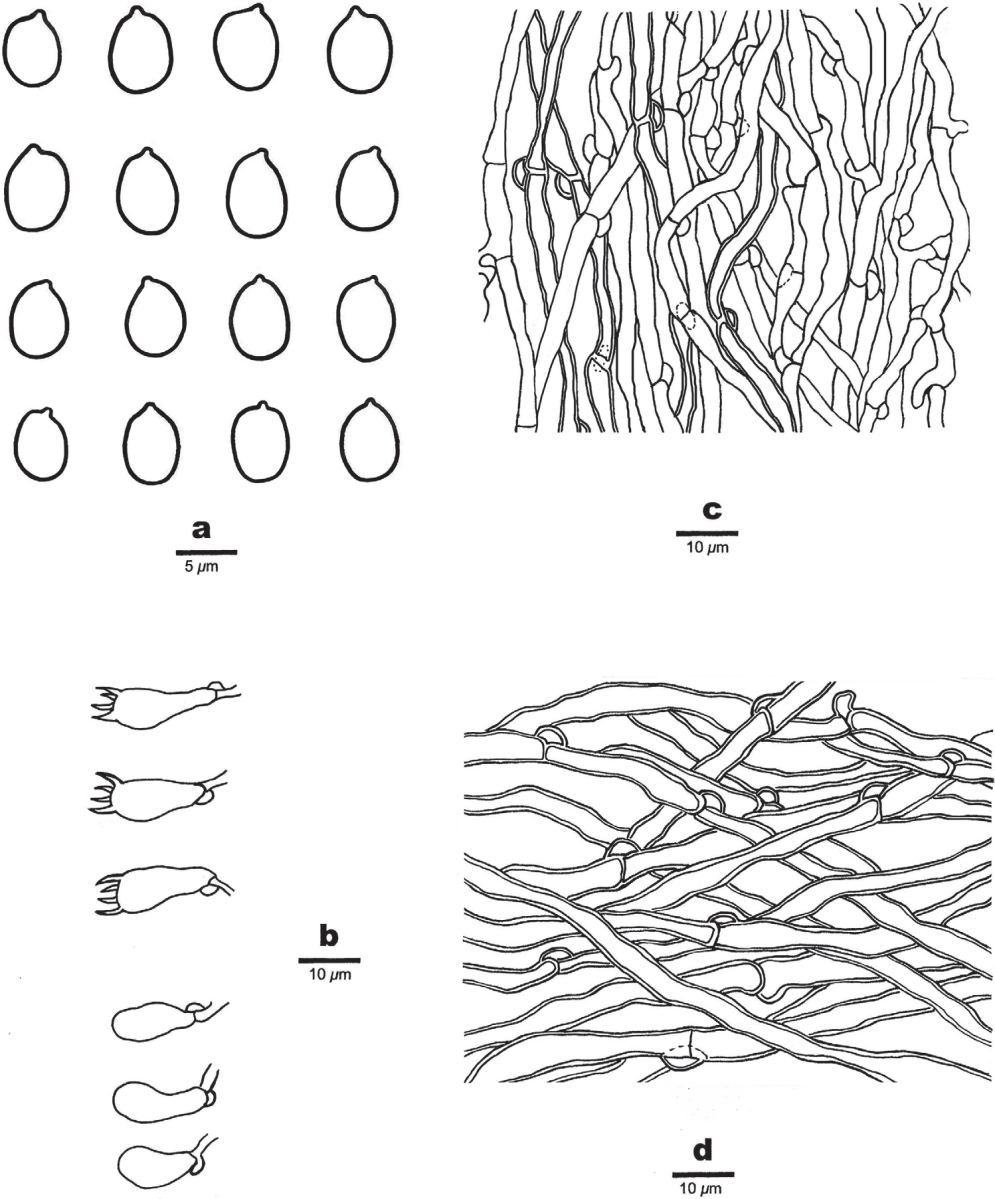
Microscopic structures of *Bjerkandera resupinata* (holotype, Y.C. Dai 16642) **a** basidiospores **b** basidia and basidioles **c** hyphae from trama **d** hyphae from subiculum.

##### Basidiospores.

Ellipsoid to broadly ellipsoid, hyaline, thin-walled, smooth, CB–, IKI–, 4.5–6(–6.2) × 3.2–4.1(–4.2) µm, L = 5.23 μm, W = 3.71 μm, Q = 1.40–1.42 (n = 60/2).

##### Additional specimen (paratype) examined.

China. Yunnan Province, Tengchong County, Gaoligong Mts., fallen angiosperm branch, 24. X. 2009, B.K. Cui 8017 (BJFC006506).

##### Remarks.

*Bjerkandera resupinata* is characterised by resupinate basidiomata, pinkish buff to pale brownish pore surface, clavate basidia, and ellipsoid to broadly ellipsoid basidiospores measuring 4.5–6 × 3.2–4.1 µm. *Ceriporiopsis umbrinescens* (Murrill) Ryvarden and *Bjerkandera resupinata* have resupinate basidiomata, pale buff to brownish pore surface, similar sterile margin, a monomitic hyphal structure, and almost the same size of basidiospores, but *C. umbrinescens* has bigger pores (2–4 per mm), unchanged pore surface when touched, and a dark line absent between tubes and subiculum (Murrill 1920; Domański 1963; Núñez and Ryvarden 2001; Zhao et al. 2015).

**Table 2.** Morphological comparison of the currently accepted species in *Bjerkandera*.

	Basidiomata type	Pilei colour	Pore shape and number of pores	Poroid surface	Basidiospores size (μm)	Basidiospores shape	Reference
***B. adusta***	Pileate, effused-reflexed to resupinate	Cream to buff, then greyish to greyish-blue	Round to angular, 6–7/mm	Grey to black	4.5–6 × 2.5–3.5	Short-cylindrical to subellipsoid	Ryvarden and Melo 2017
***B. albocinerea***	Pileate to effused-reflexed	Sordid white to pale cream	Round, 8–11/mm	Dark brown grey to almost black when bruised	3.5–4.5 × 2–2.6	Oblong-ellipsoid to ellipsoid	Motato-Vásquez et al. 2020
***B. atroalba***	Pileate to effused-reflexed	White to cream, then grey	Round or more commonly angular, 2–5/mm	White to cream, then becoming dark	4–5 × 3–4	Narrowly ellipsoid to broadly ellipsoid	Westphalen et al. 2015
***B. centroamericana***	Pileate to effused-reflexed	White to cream, then brownish	Angular, 7–11/mm	Sordid white, then brown to black in bruised parts	4–5 × 3–4.5	Broadly ellipsoid to subglobose	Westphalen et al. 2015
***B. ecuadorensis***	Pileate	Pinkish-buff to buff	Round to angular, 7–9 /mm	Grey to dark-brown, then almost black in bruised parts	3.9–4.5 × 2.7–3	Ellipsoid	Present study
***B. fulgida***	Effused-reflexed	Pinkish buff to clay-buff	Round or sometimes angular, 6–8/mm	Clay-buff to pale brown, then dark brown in bruised parts	3.9–4.5 × 2.8–3.3	Ellipsoid to broadly ellipsoid	Present study
***B. fumosa***	Pileate to effused-reflexed	Buff to woody coloured	Round to angular 2–5/mm	Buff to isabelline	5.5–7 × 2.5–3.5	Short cylindrical	Ryvarden and Melo 2017
***B. mikrofumosa***	Effused-reflexed	Pale golden-brown	Angular, 7–9/mm	Pale to smoky brown, then dark grey in bruised parts	3.5–4.8 × 2.3–3	Ellipsoid	Motato-Vásquez et al. 2020
***B. minispora***	Pileate	Pinkish-buff to buff	Round to angular, 6–9/mm	Buff-yellow, ash-grey to pale brown, then almost black in bruised parts	3.1–4.2 × 2–2.8	Oblong-ellipsoid to ellipsoid	Present study
***B. resupinata***	Resupinate	–	Round to angular, 4–6/mm	Pinkish buff to pale brownish, then dark grey in bruised parts	4.5–6 × 3.2–4.1	Ellipsoid to broadly ellipsoid	Present study

##### Additional specimens examined.

***Bjerkandera adusta***: China. Heilongjiang Province, Heihe, Shengshan Nature Reserve, *Populus*, 26. VIII. 2014, Y. C. Dai 14516 (BJFC017794); Yunnan Province, Yongde County, Daxueshan Nature Reserve, rotten Angiosperm stump, 27. VIII. 2015, Y. C. Dai 15665 (BJFC019769); Gansu Province, Tianshui, Fangmatan Forest Park, fallen branch of *Populus*, 08. VIII. 2015, Y. C. Dai 15495 (BJFC019600). France. Lyons, *Abies*, 24. XI. 2012, Y. C. Dai 13201 (BJFC014065). Finland, Helsinki, Tamisto Nature Reserve, *Betula*, 4. XI. 2011, Y. C. Dai 12640 (BJFC012222). ***B. albocinerea***: USA. CT, CAES Valley Lab, Dead log, 13. XII. 2015, Y. C. Dai 16411 (BJFC020499). ***B. fumosa***: China. Chongqing, Jinfoshan Forest Park, dead angiosperm tree, 1. XI. 2019, Y. C. Dai 21100 (BJFC032759); Beijing, Chinese Academy of Sciences, living tree of *Diospyros*, Y. C. Dai 21087 (BJFC032746); Sichuan Province, Xiaojin County, Jiajin Mts., *Hippophae*, 17. X. 2012, B. K. Cui 10747 (BJFC013669). Finland, Helsinki, Tamisto Nature Reserve, *Populus*, 6. XI. 2011, Y. C. Dai 12474B (BJFC012257). ***B. mikrofumosa***: Costa Rica. Monteverde, J. Vlasák Jr. JV 1707/10J-1; JV 1707/10J-2. ***B. centroamericana***: Costa Rica, Carara Nature Reserve, J. Vlasák JV 1704/97. ***B. atroalba***: Brazil. Recife, Charles Darwin Ecological Reserve, on angiosperm stump, Y. C. Dai 17457 (BJFC024988). ***Ceriporiopsis carnegieae***: USA, Virgin Islands, St. John, on hard wood, J. Vlasák Jr. JV 0409/27-J. ***Ceriporiopsis* sp.**: Costa Rica. Arenal Mts., J. Vlasák Jr. JV 1512/13-J.

### Key to the species of *Bjerkandera*

**Table:** 

1	Basidiomata resupinate	***B. resupinata***
–	Basidiomata effused-reflexed to pileate	**2**
2	Pores < 5 per mm	**3**
–	Pores > 5 per mm	**4**
3	Pileal surface white to cream; basidiospores broadly ellipsoid	***B. atroalba***
–	Pileal surface buff to woody-coloured; basidiospores short cylindrical	***B. fumosa***
4	Pileal surface white to cream when fresh	**5**
–	Pileal surface buff to grey when fresh	**6**
5	Basidiospores subglobose to broadly ellipsoid	***B. centroamericana***
–	Basidiospores oblong-ellipsoid to ellipsoid	***B. albocinerea***
6	Crystals present among hymenium	**7**
–	Crystals absent among hymenium	**8**
7	Pileal margin dark brown when dry	***B. mikrofumosa***
–	Pileal margin buff when dry	***B. fulgida***
8	Basidiospores > 4.5 μm in length	***B. adusta***
–	Basidiospores < 4.5 μm in length	**9**
9	Basidiospores 3.1–4.2 × 2–2.8 μm, Q = 1.49–1.53	***B. minispora***
–	Basidiospores 3.9–4.5 × 2.7–3 μm, Q = 1.43	***B. ecuadorensis***

## Discussion

Our phylogeny recovered *Bjerkandera* as a monophyletic genus, with ten species including the four new species – *Bjerkandera ecuadorensis*, *B. fulgida*, *B. minispora*, and *B. resupinata* – nested in the *Bjerkandera* clade (Fig. 1).

*Bjerkandera ecuadorensis*, *B. minispora*, *B. adusta*, *B. albocinerea*, and *B. fumosa* are phylogenetically related (Fig. 1). *B. adusta*, *B. albocinerea*, and *B. fumosa* form a group which is consistent with previous studies (Westphalen et al. 2015; Motato-Vásquez et al. 2020). The specimen we studied Dai 16411 from CT, USA and *Bjerkandera albocinerea* share cream to buff-yellow pileal surface when dry, dark brownish grey to black pore surface, round pores (8–11 per mm), and oblong-ellipsoid to ellipsoid basidiospores (3.5–4.5 × 2–2.5 μm). Also, there are two base pairs differences between them, which amounts to < 1% nucleotide differences in the ITS regions. So both specimens represent the same species. The type of *Bjerkandera albocinerea* and other specimens were collected from Brazil, but the specimen Dai 16411 from CT, USA, *B. albocinerea* has a wide distribution in America. Morphologically, *Bjerkandera albocinerea* is different from other four species by its white fresh pileal surface (Motato-Vásquez et al. 2020), and *B. minispora* can be distinguished from *B. ecuadorensis*, *B. adusta* and *B. fumosa* by smaller basidiospores (3.1–4.2 × 2–2.8 μm in *B. minispora*, 3.9–4.5 × 2.7–3 μm in *B. ecuadorensis*, and 4.5–6 × 2.5–3.5 μm in *B. adusta*, 5.5–7 × 2.5–3.5 μm in *B. fumosa*, Ryvarden and Melo 2017). *Bjerkandera fumosa* has the thicker context usually more than 6 mm, while the other two less than 6 mm (Ryvarden and Melo 2017). *Bjerkandera adusta* has short-cylindric to subellipsoid and bigger basidiospores (4.5–6 × 2.5–3.5 μm, Ryvarden and Melo 2017), which can differ from *B. ecuadorensis*. Also, there are 21 base pairs differences between *Bjerkandera ecuadorensis* and *B. minispora*, which amounts to > 2% nucleotide differences in the ITS regions.

*Bjerkandera fulgida* grouped with *B. resupinata* in a joint subclade, and these two species are closely related to *B. atroalba* (Rick) Westph. & Tomšovský, *B. centroamericana* Kout, Westph. & Tomšovský, and *B. mikrofumosa* Ryvarden with strong support (99/98/1.00). *Bjerkandera resupinata* has resupinate basidiomata, big pores and basidiospores, which can be distinguished from *B. fulgida* indeed. Also, there are eight base pairs differences between them, which amounts to 2% nucleotide differences in the ITS regions. *Bjerkandera atroalba*, *B. centroamericana* and *B. mikrofumosa* have a neotropical distribution (Westphalen et al. 2015; Motato-Vásquez et al. 2020), while *B. fulgida* and *B. resupinata* from tropical China are proved to nest in the group according to our phylogenetic study. Morphologically, *Bjerkandera resupinata* is a resupinate species, while basidiomata are effused-reflexed to pileate in *B. fulgida*, *B. atroalba*, *B. centroamericana*, and *B. mikrofumosa* (Westphalen et al. 2015; Motato-Vásquez et al. 2020). *Bjerkandera atroalba* and *B. centroamericana* differ from *B. fulgida* by their white pilei when fresh, sordid white to cream pore surface, and the presence of cystidioles (Westphalen et al. 2015). *B. mikrofumosa* differs from *B. fulgida* by its pale golden-brown pileal surface and pale to smoky brown pore surface (Motato-Vásquez et al. 2020). In addition, we found *Ceriporiopsis umbrinescens* (Murrill) Ryvarden and *B. resupinata* have resupinate basidiomata, pale buff to brownish pore surface, similar sterile margin, a monomitic hyphal structure, and almost the same size of basidiospores, but *C. umbrinescens* has bigger pores (2–4 per mm) and unchanged pore surface when touched (Murrill 1920; Domański 1963; Núñez and Ryvarden 2001; Zhao et al. 2015).

In our phylogenetic analysis, *Ceriporiopsis carnegieae* (D.V. Baxter) Gilb. & Ryvarden is phylogenetically close to the genus *Bjerkandera*. *Ceriporiopsis* Domański is a polyphyletic genus, which is nested in the families Irpicaceae, Meruliaceae (the type species *C. gilvescens* (Bres.) Domański belongs to Meruliaceae), and Phanerochaetaceae (Justo et al. 2017). Meanwhile, *Ceriporiopsis carnegieae* resembles *Bjerkandera* by having a monomitic hyphal system, generative hyphae with abundant clamps, and oblong to short-cylindric basidiospores (Baxter 1941; Gilbertson and Ryvarden 1985). However, the former has basidiomata with sharp and pungent odor when fresh, unchanged pore surface when touched or bruised, and seem to lack any dark line between tubes and subiculum (Gilbertson and Ryvarden 1985). One specimen – JV1512–13J – from Costa Rica forms a sister group to the three sequences annotated as *Ceriporiopsis carnegieae*, and we treat this specimen as *Ceriporiopsis* sp. There is ongoing controversy regarding for the generic affiliation of *C. carnegieae* (Nobles 1965; Justo et al. 2017; Motato-Vásquez et al. 2020), because the black line is absent from *Ceriporiopsis carnegieae.* For the time being, we are reluctant to combine them in *Bjerkandera* although the two taxa are phylogenetically related. To solve this problem more specimens should be examined and analysed phylogenetically.

Beside the ten species of *Bjerkandera* in our phylogeny (Fig. 1), another three taxa – *Bjerkandera terebrans* (Berk. & M.A. Curtis) Murrill, *B. subsimulans* (Berk. et M. A. Curtis) Murrill and *B. amorpha* (Fr.) P. Karst. – were included in the genus. However, *Bjerkandera terebrans* was mentioned probably as a form of *B. fumosa* or variant of *Osteina obducta* (Berk.) Donk Because of its basidiomata with a stipe-like base (Murrill 1907; Zmitrovich et al. 2016). *B. subsimulans* has lobed and broadly sterile margin with a zone of appressed hairs, and angular irregular pores (1–3 per mm), which are in accord with the description of *Abortiporus biennis* (Bull.) Singer (Murrill 1907; Zmitrovich et al. 2016). *B. amorpha* has dimitic hyphal system and allantoid basidiospores that differ from *Bjerkandera*, so it is now *Skeletocutis amorpha* (Fr.) Kotl. & Pouzar (Kotlába and Pouzar 1958).

*Tyromyces vivii* Homble ex Ryvarden was described from Norway (Ryvarden et al. 2003), and later it was treated as a synonym of *B. fumosa* (Ryvarden and Melo 2017). The type material of *T. vivii* was analyzed, and it nested in *B. fumosa* (Fig. 1). We confirm this conclusion by molecular evidence.

Previously, the well-known *Bjerkandera adusta* and *B. fumosa* have been reported from the northern hemisphere and South America. However, the diversity of *Bjerkandera* was underestimated, *B. centroamericana*, *B. mikrofumosa* and *B. albocinerea* were recently described in the neotropics (Westphalen et al. 2015; Ryvarden 2016), and new species in our study have a distribution in the neotropics and tropical Asia. So, the genus has a wide distribution from boreal to tropical areas.

## Supplementary Material

XML Treatment for Bjerkandera ecuadorensis

XML Treatment for Bjerkandera fulgida

XML Treatment for Bjerkandera minispora

XML Treatment for Bjerkandera resupinata
